# High Recurrence Rate of Myxofibrosarcoma: The Effect of Radiotherapy Is Not Clear

**DOI:** 10.1155/2019/8517371

**Published:** 2019-10-01

**Authors:** Hjalmar Teurneau, Jacob Engellau, Iman Ghanei, Fredrik Vult von Steyern, Emelie Styring

**Affiliations:** ^1^Lund University, Skane University Hospital, Department of Orthopedics, Lund, Sweden; ^2^Lund University, Skane University Hospital, Department of Oncology, Lund, Sweden

## Abstract

**Background:**

Myxofibrosarcoma (MFS) is one of the more common types of soft-tissue sarcoma (STS) in patients over 60 years of age. Local recurrence (LR) rates have been reported to be higher compared to other STS types.

**Patients and Methods:**

Using a population-based series from the southern Sweden health care region, 56 consecutive patients with MFS and localized disease at diagnosis were analyzed with respect to LR and distant metastases after surgery ± adjuvant treatment.

**Results:**

The overall local recurrence (*n* = 15) and metastasis (*n* = 13) rates were 27% and 21%, respectively; 6 patients had both. Surgical margin was the only statistically significant prognostic factor for LR. Patients operated with a marginal margin had an HR of 4.5 (CI 1.3–15.1, *p*=0.02) and those operated with an intralesional margin 9.4 (CI 2.0–43.5, *p*=0.004) compared to those operated with a wide surgical margin. There was no difference in the LR rate depending on radiotherapy or not, although the latter group had smaller and more superficial tumors. 23 patients received radiotherapy, 9 of whom developed LR, all within the irradiated field. A tumor size >5 cm and intralesional surgical margin were shown to be risk factors for distant metastases.

**Conclusions:**

The rate of LR for patients with myxofibrosarcoma was high. The impact of RT on local tumor control was unclear. The surgical margin was important for both local and distant tumor control. Large tumor size was a risk factor for distant metastasis.

## 1. Introduction

Myxofibrosarcomas (MFSs) are malignant soft-tissue tumors typically presenting as a slowly growing, painless mass in the extremities or the superficial trunk. They most commonly occur in patients between 60 and 70 years of age, with a slight predominance in males. Historically, MFS was described as a kind of malignant fibrous histiocytoma (MFH) but has gradually been recognized as a distinct histotype. The histopathologic patterns of myxofibrosarcoma are characterized by a myxoid component of extracellular matrix, pleomorphic spindle cells, and curvilinear blood vessels. There are no specific immunohistochemical markers or genetic profiles for MFS, but the techniques are useful in excluding similar but differential tumors. Superficial MFSs [1] often consist of multiple palpable nodules, while the deep-seated lesions more often form a single mass. The tumors have a peripheral infiltrative growth pattern with extension along vascular and fascial planes extra- or intramuscularly [1]. In this study, the peripheral growth pattern was evaluated microscopically and classified as infiltrative if signs of infiltrative growth into the surrounding tissue could be seen [2]. Tumor necrosis is typically found in the high-grade but not in the low-grade MFS [3].

MFS has a reported LR rate of 17–54% which is high compared to other soft-tissue sarcomas [1, 3–11]. No correlation between the MFS malignancy grade and the rate of LR has been reported [3, 7, 8]. Rather, the increased rate is considered because of its infiltrative peripheral growth pattern. Defining the tumor demarcation is difficult in the preoperative setting, using magnetic resonance imaging (MRI) and computerized axial tomography (CAT), as well as during surgery, thus increasing the risk of insufficient surgical margin [12]. The tumors in this series were graded using the four-tiered Brodie system where grades 1 and 2 are low-grade tumors and grades 3 and 4 high-grade tumors [13]. Different tumor malignancy grading systems have been used over the years. Overall, the malignancy grade correlates with the rate of metastases and tumor-related death. For example, grade 2 and 3 MFSs (according to the three-tiered FNCLCC system) are reported to develop metastases in 15 to 35% of cases compared to none of the low-grade MFS [3, 8].

To identify high-grade STSs with a high risk of developing metastases, we use the SING prognostic system evaluating size (>8 cm = 1p), vascular invasion (present = 2p), necrosis (present = 1p), and peripheral growth pattern (present = 1p) in Scandinavia [2, 14–17]. Patients whose tumors have at least 2 points according to the SING model are considered for chemotherapy.

Surgical margin was defined according to the Scandinavian Sarcoma Group's definition [17, 18]. Thus, it was classified as intralesional if microscopic tumor tissue was seen at the resection border or if tumor rupture occurred during surgery. Tumor-free (R0) margins were defined as wide if there was a cuff of healthy tissue all around the tumor, either an unengaged fascia or at least 10 mm of fatty, muscular, or loose areolar tissue. Otherwise, they were classified as marginal margins. To improve local control, all deep-seated, high-grade soft-tissue sarcomas, regardless of the surgical margin, and all high-grade STSs following marginal and intralesional margins, irrespective of tumor depth, are recommended adjuvant RT [18]. The most common RT courses used in Scandinavia for soft-tissue sarcomas of the extremity and trunk wall with adequate surgical margins (wide or marginal) are 50 Gy in 25 fractions or 36 Gy hyperfractionated with 1.8 Gy twice daily when combined with chemotherapy. Intralesional surgical margins are treated with higher doses of 64–66 Gy, 2 Gy/fraction, or 45 Gy with hyperfractionation [6, 17–19].

This study was aimed to describe the rate of LR and metastasis of MFS in a population-based series of myxofibrosarcoma patients treated at the Lund sarcoma center in the southern Sweden health care region. Furthermore, we tried to identify possible independent tumor- and treatment-related risk factors for LR and metastasis.

## 2. Patients and Methods

### 2.1. Patient Cohort

All patients with MFS of the extremity or trunk wall within the southern Sweden health care region were identified using the population-based Scandinavian Sarcoma Group's register [16]. In total, 56 patients with localized MFS were diagnosed and operated between October 15, 1998, and October 2016. The median age at presentation was 72 years (range 35 years–95 years), and 33 patients were male.

### 2.2. Tumor Characteristics

Forty-six of 56 tumors in this series were of high grade (Table 1). Size was determined as the largest diameter at the pathology review. Vascular invasion and necrosis were classified as present or absent and the peripheral growth pattern as infiltrative or pushing at the pathology review. 31 tumors had necrosis; 7 vascular invasions and all 37 tumors, where they were assessed, had infiltrative margins (Table 1).

Tumors were classified as superficial if they were strictly subcutaneous, i.e., did not involve the fascia. Otherwise, they were classified as deep-seated. The tumor site was defined as the upper or lower extremity or, as in a few cases, the trunk wall.

### 2.3. Referral and Treatment Factors

The referral pattern was assessed as virgin before any invasive procedure (*n* = 37), after fine needle or core biopsy (*n* = 7), after excision (*n* = 11), or after local recurrence (*n* = 1). 46 patients underwent one surgery, while 10 patients had two operations for their primary tumor (i.e., the second surgery within three months of the first). The surgical margin was determined in agreement between the surgeon and the pathologist. For a margin to be classified as wide, an unengaged fascia or a 10 mm cuff of healthy fatty, muscular, or loose areolar tissue was necessary. Less than 10 mm margin or an engaged fascia was defined as a marginal margin, and tumor growth at the resection margin was defined as an intralesional margin.

Radiotherapy was defined as administered or not, and data on fractioning and dose/fraction were recorded. Chemotherapy was defined as administered or not without further analysis of used drugs or doses.

### 2.4. Follow-Up and Outcome Parameters

Patients were routinely followed up for 10 years with physical examination of the primary tumor area and a chest X-ray at specified intervals (depending on time from diagnosis and tumor malignancy grade). MRI of the primary tumor site was performed if an LR was suspected or the physical examination was difficult to evaluate. The median follow-up time was 5.1 years (range 7 months–16.5 years).

The study end points were local recurrence and distant metastasis (analyzed separately).

### 2.5. Data Collection and Statistical Analysis

The register data were prospectively recorded and validated through retrospective examination of clinical records. Additional treatment data and clinical outcomes were retrieved.

An in-depth analysis of preoperative MRI of the primary tumor, radiotherapy fields, and LR locations was performed to determine if LR occurred within or outside the irradiated field.

Based on previous studies, potential risk factors for LR and metastasis were tested using the log-rank test for univariate analysis. To estimate hazard ratios and for multivariate analysis, Cox regression was performed using the surgical margin as a factor on 3 levels.

The proportion of patients free of LR and metastasis vs. time since diagnosis was estimated and plotted using the Kaplan–Meier method. Patients were censored from the analysis at the end of follow-up, if they had no evidence of disease or had died without tumor.

A two-sided *p* value <0.05 was considered statistically significant. All statistical analyses were performed using STATA 12 (StataCorp LP, College Station, TX).

## 3. Results

### 3.1. Surgical Treatment

In total, 46 patients underwent one surgery, 42 of whom had their primary surgery at the sarcoma center. In 8 cases, the primary surgery was performed outside the sarcoma center. The 10 patients who had 2 surgeries for primary tumor all had the second surgery performed at the sarcoma center. The final surgical margins, after one or two surgeries for the primary tumor, were wide in 30 patients, marginal in 19 patients, and intralesional in 7 patients.

The 12 patients undergoing their first surgery outside of the sarcoma center had a median tumor size of 4.5 cm, 11 had superficial tumors, and 1 had a deep-seated tumor. After the first surgery, 8/12 patients had intralesional surgical margins, 3 had marginal margins, and 1 had wide margins.

For the 44 patients who underwent primary surgery at the sarcoma center, the median tumor size was 8 cm; 15 patients had subcutaneous tumors, and 29 had deep-seated tumors. 3 had intralesional margins, 19 had marginal surgical margins, and 22 had wide surgical margins.

### 3.2. Adjuvant Therapy

In addition to surgery, 23 patients had adjuvant radiotherapy (Table 2), 11 had both adjuvant chemotherapy and radiotherapy, and 1 had adjuvant chemotherapy. 21 patients were treated by surgery alone. A description of patients, treatment, and tumor data in addition to outcomes is presented in Supplementary Table 1 (available here). Postoperative radiotherapy was thus administered to 34/56 patients at a median of 3.1 months (range 1 months–8 months) after surgery. No patient was treated with neoadjuvant radiotherapy.

Nineteen patients received 50 Gy in 25 fractions (one with a boost to 66 Gy; see below), eleven received 36 Gy hyperfractionated with 1.8 Gy twice daily, two received 36 Gy in 12 fractions, and one received 30 Gy in 10 fractions. One patient could not complete the intended treatment (50 Gy/25fr) and received 18 Gy in 9 fractions.

Of the 7 patients who had a final intralesional surgical margin, 3 had RT. One patient (case no. 32; Supplementary [Supplementary-material supplementary-material-1]) received a boost to 66 Gy towards the intralesional area. The second patient (case no. 48; Supplementary [Supplementary-material supplementary-material-1]) received the standard dose of 50 Gy because of a grade 2 tumor with a complex genetic profile. A reoperation would have been done to mutilating why RT was given. The third patient (case no. 46; Supplementary [Supplementary-material supplementary-material-1]) was treated with a shorter RT course, 36 Gy/12 fractions, because of comorbidities. The four remaining patients had severe comorbidities to benefit from RT.

Twelve patients with high-grade tumors received chemotherapy, 11 of whom also received radiotherapy.

### 3.3. Local Recurrence

**Figure 1:**
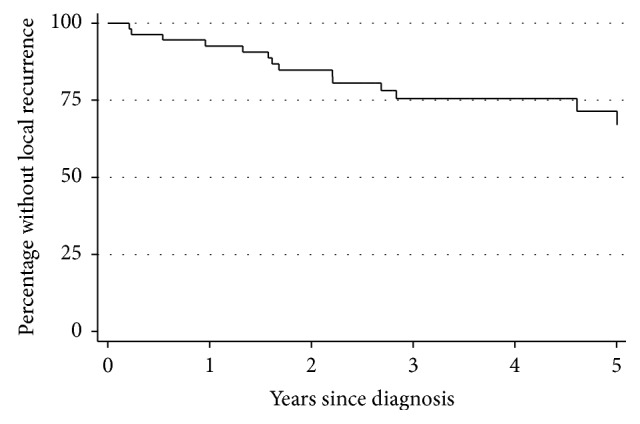
Kaplan–Meier curve illustrating time to local recurrence among all MFSs.

Fifteen out of fifty-six (27%) patients developed an LR at a median of 20 months (range 2 months–11 years; Figure 1). Intralesional (HR 9.4, 95% CI 2.0–43.5, *p*=0.004) and marginal (HR 4.5, 95% CI 1.3–15.1, *p*=0.02) final surgical margins had an increased risk of LR compared to a final wide surgical margin (Table 3).

Five out of twelve (33%) patients referred after surgery developed an LR compared to 10/44 (14%) patients referred before surgery.

Among the patients who received RT, 9/34 (27%) had LR, while 6/22 (27%) patients that did not receive RT developed LR. All 9 patients who developed LR after radiotherapy did so within the irradiated field.

No tumors showed a pushing growth pattern in this series. In all 37 tumors where the peripheral growth pattern was assessed, the peripheral growth pattern was infiltrative; 11 patients with these tumors had LR. The growth pattern was not determined in 19 cases, 4 of whom had LR. Three of these 4 LRs occurred in patients with superficial tumors <5 cm who underwent their first surgery outside the sarcoma center.

Other analyzed factors were not associated with risk for local recurrence on univariate analysis (Table 3).

### 3.4. Metastasis

**Figure 2:**
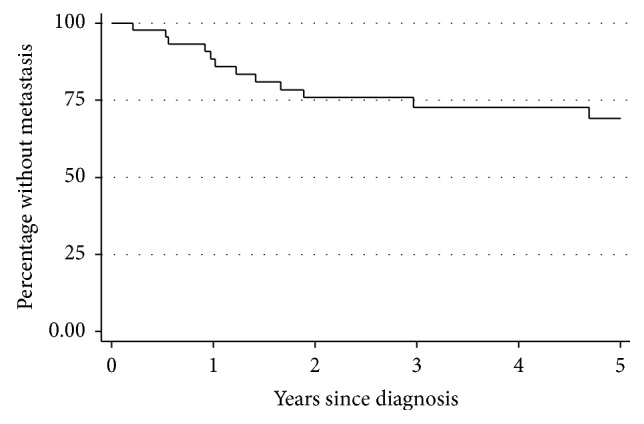
Kaplan–Meier curve illustrating time to metastasis among high-grade MFSs.

Distant metastatic disease occurred in 13/46 (28%) patients with high-grade tumors, at a median of 14.7 months after diagnosis (range 2.5 months–7.7 years; Figure 2), including 6 patients who also developed local recurrences. Since no patients with low-grade tumors developed metastases, they were excluded from further risk factor analysis.

The median tumor size was 11 cm (range 5 cm–18 cm) in patients who did develop metastases and 6 cm (range 2 cm–17 cm) in those who did not develop metastases (Table 4). When applying a cutoff of 5 and 8 cm, there was a statistically significant difference between those who developed metastases and those who did not (log-rank test: *p*=0.02 and *p*=0.01, respectively).

Ten out of thirty (33%) tumors with tumor necrosis and 2/13 (15%) tumors without tumor necrosis metastasized. Tumor necrosis was not a statistically significant risk factor for metastasis. 2/7 (28%) tumors with vascular invasion and 9/34 (26%) tumors without vascular invasion metastasized; no statistical correlation was found.

An intralesional final surgical margin increased the risk of distant metastasis compared to a wide surgical margin (HR 6.1, 95% CI 1.0–37, *p*=0.05). There was also a trend towards a difference between wide and marginal surgical margins (HR 2.8, 95% CI 0.9–13, *p*=0.07).

Four out of twelve (33%) patients treated with chemotherapy developed distant metastases versus 9/34 (26%) patients not treated with chemotherapy.

## 4. Discussion

### 4.1. Local Recurrence Rate

We found that 15/56 (27%) patients with MFS developed an LR. This is in concordance with previous studies presenting 5-year LR rates of 17–54% [1, 3–9, 20]. Our results indicate a rather high rate of LR compared to a mixed series of STSs where the LR rate has been reported to be 15–20% [10, 11]. Local recurrences were observed to occur late, in 2 cases more than 5 years after diagnosis.

### 4.2. Risk Factors for Local Recurrence

As expected, a wide surgical margin improved the local tumor control compared to marginal and intralesional margins. In this series, we could not demonstrate improved tumor control by administration of radio- and/or chemotherapy.

The local recurrence rate in the group given RT was 9/34 (27%) compared with 6/22 (27%) in those not receiving RT. However, patients who underwent RT were more likely to have high-grade, deep-seated tumors, which are >5 cm in size and excised with intralesional or marginal margins. Since these are known risk factors for local recurrence, one may expect a higher rate of local recurrence in this group compared to the non-RT group [6, 8, 9, 19, 21]. We therefore hypothesize that the similar LR rates between the RT group and the non-RT group suggest that radiotherapy had an effect, although not statistically proven in this series. Also, Hong et al. and Haglund et al. [7, 22] describe larger tumors with more risk factors but similar LR rates when comparing the RT group to the non-RT group.

### 4.3. Radiotherapy Dose, Fraction, and Timing

Interestingly, the 9 patients who developed LR after RT all had in-field recurrences. Hence, the RT-field margins seem to be sufficient, but the radiation dose and/or fractioning may have been insufficient. Haglund et al. described LRs within the irradiated field, as in our series, but after adjuvant RT of total doses of 60–66 Gy with 1.8–2.0 Gy/fraction [7]. Because of the low incidence and multitude of histotypes in STS, it is difficult to determine radiosensitivity of specific sarcoma entities. The results in our series suggest that the current margins used are adequate, but the RT dose is perhaps insufficient. In fact, in this retrospective analysis, the radiotherapy given was not fully in accordance with our current guidelines, where intralesional margins are basis for the higher RT dose of 66 Gy/33fr, or 45 Gy/25fr with hyperfractionation. This may explain the indicated efficacy of RT to partly compensate for inadequate surgical margins in this series. The patients who received individual, out-of-protocol radiotherapy regimes were all very old, with a median age of 90 years, which reflects the inherent difficulty in achieving a homogeneous treatment series in sarcoma populations, despite clear treatment guidelines.

All the patients in this series received adjuvant RT. In other studies of MFS, RT has been administered either as neoadjuvant or adjuvant treatment or both. To our knowledge, no study comparing neoadjuvant to adjuvant RT for MFS has been published. When comparing studies of MFS, we have not found any systematic differences in the reported LR rates with respect to the timing of RT [8, 9, 20, 22–24].

### 4.4. Metastasis

The overall metastasis rate (23%) was comparable to what has been previously reported [7–9, 20]. Among high-grade tumors, 13/46 (28%) metastasized, and an intralesional surgical margin increased the risk of metastases compared to a wide surgical margin (*p* < 0.05). This suggests that surgical margins matter for both local and distant control as was described by Boughzala-Bennadji et al. [20].

In Scandinavia, tumors with a high risk of metastasis are identified based on the SING criteria (size ≥8 cm and presence of vascular invasion, necrosis, and/or peripheral growth pattern) [2, 14–17]. In this series, larger tumors had an increased risk of metastasis regardless of cutoff (5 or 8 cm), which confirms previously published results [20]. However, neither necrosis nor vascular invasion is a statistically significant risk factor for metastasis in this series. Furthermore, the growth pattern could not be analyzed since there were no tumors with pushing peripheral growth.

Patients treated with chemotherapy did indeed have a higher distant metastasis rate, despite chemotherapy, suggesting correct identification of high-risk tumors. The benefits of chemotherapy in MFS are not fully investigated, and similar to radiosensitivity, chemosensitivity is difficult to determine in these rare entities.

The diversity of STS subtypes often causes different entities to be merged to increase quantity of samples in studies. Thus, many prognostic factors of general importance for distant metastases in STS have been recognized. However, if they are adequate for each subtype remains unclear. Here, we can confirm the importance of tumor size and wide surgical margins for prognosis of metastasis-free survival in MFS patients.

### 4.5. Limitations of the Study

This study represents patients treated at a single institution as such, and the results may be influenced by treatment traditions at the sarcoma center. However, this study is population based and includes patients regardless of their referral status, socioeconomic factors, and age. This study spans over a long period of time, during which the treatment protocols may have changed. The limited number of cases is a problem in studies of rare tumor entities, and the size of our series is about the same as that in other previously published studies on MFS.

## 5. Conclusion

This series of 56 patients with MFS treated with local excision in the southern Sweden health care region showed an overall LR rate of 27%. We could not see any obvious effect of RT on local control. However, since high-risk tumors were the ones treated with RT, it can be argued that the similar LR rate in the RT and non-RT groups implies an effect. The fact that all 9 patients who developed an LR after RT had in-field recurrences suggests that MFS may require higher RT doses and/or hyperfractionated RT rather than larger fields.

The distant metastasis rate among high-grade tumors was 28%. Larger tumors were more likely to develop metastases. A wide surgical margin was associated with a better outcome with respect to both local and distant tumor control.

## Figures and Tables

**Table 1:** Overview of key tumor characteristics.

	Low grade (*n* = 10)	High grade (*n* = 46)
Tumor size (cm) (median (range))	3 (2–10)	7 (2–18)
Tumor site		
Upper extremity	1	16
Lower extremity	8	28
Trunk wall	1	2
Tumor depth		
Subcutaneous	7	19
Deep	3	27
Necrosis		
Yes	1	30
No	7	13
Not determined	2	3
Vascular invasion		
Yes	—	7
No	8	34
Not determined	2	5
Growth pattern		
Infiltrative	7	30
Pushing	—	—
Not determined	3	16

**Table 2:** Tumor and treatment factors with respect to the number of patients receiving radiotherapy (RT) and local recurrence (LR).

	No RT (LR), *n*	RT (LR), *n*
Median tumor size (range; cm)	4.5 (2–17)	8 (2–18)
Tumor depth		
Subcutaneous	15 (5)	11 (2)
Deep	7 (1)	23 (7)
Place where the primary surgery was performed		
Sarcoma center	17 (3)	27 (7)
Outside of the center	5 (3)	7 (2)
Final surgical margin^*∗*^		
Intralesional	4 (1)	3 (2)
Marginal	4 (2)	15 (6)
Wide	14 (3)	16 (1)
Additional adjuvant therapy		
None	21 (6)	23 (6)
CT	1 (0)	11 (3)
Total RT dose		
50 Gy^*∗∗*^		19 (5)
36 Gy		13 (4)
30 Gy		1 (0)
18 Gy		1 (0)
Missing RT data		1 (0)

^*∗*^After reexcision in 10 cases. ^*∗∗*^One patient had a boost to 66 Gy.

**Table 3:** Univariate analysis of risk factors for local recurrence in all tumors.

	No LR	LR	*p* value (log-rank test)	HR for LR	95% CI (Cox regression)	*p*
Median tumor size (range; cm)	6 (2–18)	8 (3–17)	0.4			
>5 cm	26	11				
<5 cm	15	4				
Tumor site			0.5			
Upper extremity	12	5				
Lower extremity	26	10				
Trunk wall	3	0				
Tumor depth			0.7			
Subcutaneous	19	7				
Deep	22	8				
Place where the primary surgery was performed			0.2			
Sarcoma center	34	10				
Outside of the center	7	5				
Final surgical margin						
Intralesional	4	3		9.4	2.0–43.5	**0.004**
Marginal	11	8		4.5	1.3–15.1	**0.02**
Wide	26	4		—	—	—
Necrosis			0.3			
Yes	21	10				
No	16	4				
Not determined	4	1				
Vascular invasion			0.6			
Yes	6	1				
No	31	11				
Not determined	4	3				
Growth pattern			N/A			
Infiltrative	26	11				
Pushing	0	0				
Not determined	15	4				
Malignancy grade			0.1			
High grade	32	14				
Low grade	9	1				
Adjuvant treatment						
None	15	6		—	—	—
Radiotherapy	17	6		0.7	0.2–2.3	0.6
Chemotherapy	1	0		N/A		
Radio- and chemotherapy	8	3		1.0	0.2–3.9	1.0

**Table 4:** Univariate analysis of risk factors for metastasis among high-grade tumors.

	No metastasis	Metastasis	*p* value (log-rank test)	HR for LR (Cox regression)	95% CI	*p*
Median tumor size (range; cm)	6.0 (2–17)	11.0 (5–18)	**0.02**			
>5 cm	22	13				
<5 cm	11	0				
Tumor site			0.6			
Upper extremity	12	4				
Lower extremity	19	9				
Trunk wall	2	0				
Tumor depth			0.6			
Subcutaneous	15	4				
Deep	18	9				
Place where the primary surgery was performed			0.6			
Sarcoma center	25	11				
Outside of the center	8	2				
Final surgical margin						
Intralesional	4	2		6.4	1.1–39	**0.04**
Marginal	11	8		2.8	0.9–13	0.07
Wide	18	3		—	—	—
Necrosis			0.2			
Yes	20	10				
No	11	2				
Not determined	2	1				
Vascular invasion			0.5			
Yes	5	2				
No	25	9				
Not determined	3	2				
Growth pattern			N/A			
Infiltrative	22	8				
Pushing	0	0				
Not determined	11	5				
Adjuvant treatment						
None	10	3		—	—	—
Radiotherapy	15	6		0.8	0.2–3.3	0.8
Chemotherapy	0	1		1.7	0.2–16	0.7
Radio- and chemotherapy	8	3		0.9	0.2–4.5	0.9

## Data Availability

Data are available from the National Quality Register for Patients with Sarcoma, CPUA Region Skane, for researchers who meet the criteria for access to confidential data.

## Supplementary Materials

Supplementary Materials Supplementary Table 1: a description of patient-, tumor-, and treatment-related factors and outcomes.
