# Supplementary material for: High Recurrence Rate of Myxofibrosarcoma: The Effect of Radiotherapy Is Not Clear
Source: Sarcoma. 2019 Oct 1;2019:8517371. doi: 10.1155/2019/8517371 (PMC6791216; doi:10.1155/2019/8517371)
Supplement: Supplementary Materials — Supplementary Table 1: a description of patient-, tumor-, and treatment-related factors and outcomes. [file 8517371.f1.docx]

| Case no | Year of diagnosis | Sex | Age at diagnosis | First surgery at | Adjuvant treatment | Total Gy/ Fractions | Tumor size | Necrosis | Vascular invasion | Infiltrative growth pattern | Malignancy grade | LR | Metastasis | Diseased |
| --- | --- | --- | --- | --- | --- | --- | --- | --- | --- | --- | --- | --- | --- | --- |
| 1 | 1998 | Male | 76 | S.C. | None |  | 9 | - | - | NA | 3 | - | + | Yes |
| 2 | 2000 | Male | 75 | Outside S.C. | None |  | 4 | + |  | NA | 4 | + | - | Yes |
| 3 | 2000 | Male | 66 | S.C. | RT | 50/25 | 15 | + | - | NA | 4 | - | - | No |
| 4 | 2001 | Female | 64 | S.C. | RT + Chemo | 36/20 | 11 | + | - | + | 4 | - | + | Yes |
| 5 | 2001 | Male | 56 | S.C. | RT | 50/25 | 4 | - | - | NA | 4 | - | - | No |
| 6 | 2001 | Female | 78 | S.C. | None |  | 3 | NA | NA | NA | 2 | - | - | No |
| 7 | 2002 | Male | 87 | S.C. | None |  | 17 | + | + | NA | 4 | + | - | Yes |
| 8 | 2002 | Female | 85 | S.C. | RT | 18/9 | 10 | - | - | NA | 2 | - | - | Yes |
| 9 | 2002 | Female | 77 | S.C. | None |  | 7 | - | - | NA | 3 | - | - | Yes |
| 10 | 2002 | Female | 75 | S.C. | None |  | 10 | - | - | NA | 4 | - | - | Yes |
| 11 | 2002 | Female | 65 | S.C. | RT + Chemo | 36/20 | 7 | + | + | + | 3 | - | - | No |
| 12 | 2002 | Male | 54 | Outside S.C. | RT | 50/25 | 6 | NA | NA | NA | 4 | - | - | No |
| 13 | 2003 | Female | 92 | S.C. | RT | 50/25 | 13 | + | + | NA | 4 | - | + | Yes |
| 14 | 2003 | Female | 90 | Outside S.C. | RT | 36/20 | 10 | + | - | NA | 3 | + | + | Yes |
| 15 | 2003 | Male | 70 | Outside S.C. | RT | 50/25 | 3 | + | - | NA | 4 | - | - | Yes |
| 16 | 2003 | Female | 35 | S.C. | RT + Chemo | 36/20 | 9 | + | - | + | 4 | - | - | No |
| 17 | 2004 | Female | 92 | S.C. | RT | 30/10 | 8 | + | + | NA | 4 | - | - | Yes |
| 18 | 2004 | Male | 81 | Outside S.C. | None |  | 5 | NA | NA | NA | 4 | + | + | Yes |
| 19 | 2004 | Male | 71 | S.C. | RT | 50/25 | 16 | + | NA | + | 4 | + | + | Yes |
| 20 | 2004 | Male | 83 | S.C. | RT | 50/25 | 18 | + | - | NA | 4 | - | + | Yes |
| 21 | 2004 | Male | 78 | S.C. | None |  | 2 | - | - | + | 2 | - | - | Yes |
| 22 | 2004 | Female | 81 | S.C. | None |  | 8 | - | - | + | 2 | - | - | No |
| 23 | 2004 | Male | 81 | S.C. | RT | 50/25 | 5 | + | + | NA | 4 | - | - | No |
| 24 | 2004 | Male | 60 | Outside S.C. | RT + Chemo | 36/20 | 4 | + | + | + | 3 | - | - | No |
| 25 | 2005 | Female | 95 | S.C. | None |  | 6 | - | - | + | 4 | - | - | Yes |
| 26 | 2005 | Male | 56 | Outside S.C. | None |  | 3 | + | - | + | 2 | + | - | No |
| 27 | 2005 | Female | 56 | S.C. | RT | 50/25 | 2 | + | - | + | 4 | - | - | No |
| 28 | 2007 | Male | 84 | S.C. | None |  | 7 | + | + | + | 4 | - | + | Yes |
| 29 | 2007 | Male | 63 | S.C. | None |  | 4 | - | - | + | 3 | + | - | No |
| 30 | 2007 | Male | 61 | S.C. | RT | 50/25 | 15 | - | - | + | 3 | - | - | No |
| 31 | 2007 | Female | 38 | S.C. | RT + Chemo | 36/20 | 11 | + | - | + | 4 | - | - | No |
| 32 | 2008 | Male | 74 | Outside S.C. | RT | 50/25* | 4 | - | - | + | 3 | + | - | No |
| 33 | 2008 | Male | 72 | S.C. | None |  | 6 | - | - | + | 3 | + | - | No |
| 34 | 2008 | Female | 65 | S.C. | None |  | 4 | - | - | + | 2 | - | - | No |
| 35 | 2009 | Male | 72 | S.C. | RT + Chemo | 36/20 | 15 | + | - | + | 4 | + | + | Yes |
| 36 | 2009 | Male | 51 | S.C. | RT | 50/25 | 5 | - | - | + | 3 | + | + | No |
| 37 | 2009 | Female | 66 | S.C. | None |  | 2 | - | - | + | 2 | - | - | No |
| 38 | 2010 | Female | 43 | S.C. | Chemo |  | 12 | + | - | + | 4 | - | + | Yes |
| 39 | 2010 | Male | 51 | S.C. | RT + Chemo | 36/20 | 9 | + | - | + | 4 | + | - | No |
| 40 | 2010 | Male | 60 | S.C. | RT + Chemo | 36/20 | 14 | - | - | + | 4 | - | - | No |
| 41 | 2010 | Male | 77 | S.C. | RT | 50/25 | 6 | + | - | + | 4 | - | - | No |
| 42 | 2010 | Male | 71 | S.C. | None |  | 3 | NA | NA | NA | 1 | - | - | No |
| 43 | 2011 | Male | 65 | S.C. | RT | 50/25 | 8 | + | - | + | 4 | + | + | No |
| 44 | 2011 | Female | 78 | S.C. | RT | 50/25 | 11 | + | - | + | 3 | + | - | No |
| 45 | 2011 | Female | 63 | S.C. | RT + Chemo | 36/20 | 3 | + | - | + | 3 | - | - | No |
| 46 | 2012 | Male | 90 | Outside S.C. | RT | 36/12 | 5 | + | - | + | 3 | - | - | Yes |
| 47 | 2012 | Male | 37 | S.C. | RT + Chemo | 36/20 | 12 | + | - | + | 4 | - | + | No |
| 48 | 2012 | Female | 77 | S.C. | RT | 50/25 | 4 | - | - | + | 2 | - | - | No |
| 49 | 2012 | Male | 50 | Outside S.C. | None |  | 3 | - | - | + | 2 | - | - | No |
| 50 | 2013 | Male | 88 | Outside S.C. | None |  | 6 | - | - | + | 3 | - | - | Yes |
| 51 | 2013 | Female | 89 | S.C. | None |  | 4 | NA | NA | NA | 4 | - | - | Yes |
| 52 | 2013 | Female | 71 | S.C. | RT + Chemo | 36/20 | 13 | + | - | + | 4 | + | - | No |
| 53 | 2013 | Male | 88 | S.C. | RT | 50/25 | 8 | + | - | + | 4 | - | - | No |
| 54 | 2013 | Female | 75 | Outside S.C. | RT | 50/25 | 6 | + | - | + | 4 | - | - | No |
| 55 | 2014 | Male | 77 | S.C. | RT | 50/25 | 4 | + | - | + | 3 | - | - | No |
| 56 | 2014 | Male | 70 | S.C. | None |  | 2 | - | - | + |  | - | - | No |

Abbreviations: S.C. Sarcoma Center; RT radiotherapy; Chemo chemotherapy; NA not available; + Yes; - No.
* Boost to 66 Gy due to intralesional surgical margin.
